# Compartment-specific GLUT1 patterns in colorectal liver metastases: invasive-margin GLUT1 associates with outcome in solitary disease

**DOI:** 10.1007/s00262-026-04378-z

**Published:** 2026-04-06

**Authors:** Eva Grießhammer, Niklas Bogovic, Edward K. Geissler, Katja Evert, Markus Götz, Christina Hackl, Stefan Fichtner-Feigl, Hans J. Schlitt, Stefan M. Brunner

**Affiliations:** 1 Department of Surgery, University Hospital Regensburg, Franz-Josef-Strauss-Allee 11, 93053 Regensburg, Germany; 2 Department of General and Visceral Surgery, University of Freiburg, Freiburg, Germany; 3 Institute of Pathology, University of Regensburg, Regensburg, Germany

**Keywords:** Colorectal liver metastases, GLUT1, Immunometabolism, CD8+ T cells, Tumor microenvironment

## Abstract

**Background:**

Colorectal liver metastases (CRLM) are a major cause of cancer-related deaths. Glucose transporter 1 (GLUT1) is a key mediator of glycolytic metabolism in malignant and immune cells; however, the prognostic relevance of compartment-specific GLUT1 patterns in CRLM remains undefined. We hypothesized that spatial GLUT1 expression at the tumor–liver interface may reflect clinically relevant microenvironmental biology.

**Methods:**

We retrospectively analyzed data of 192 patients who underwent curative-intent resection for CRLM (75 solitary; 117 multiple). GLUT1 expression was assessed by immunohistochemistry in the tumor tissue (Tu) and infiltration margin (Im) and was correlated with overall survival using Cox regression. Double immunofluorescence for CD8 and GLUT1 was performed for qualitative visualization at the infiltration margin. In an exploratory subset (n = 5), flow cytometry and in vitro killing assays were conducted on GLUT1⁺ versus GLUT1⁻ CD8⁺ tumor-infiltrating lymphocytes (TILs).

**Results:**

Tumoral GLUT1 correlated with Ki67 (Spearman’s ρ = 0.31, p = 0.003) but was not independently associated with overall survival in the main cohort (multivariable HR 1.183, 95% CI 0.74–1.90; *p* = 0.485). In the main cohort, capsule presence remained strongly associated with improved survival (HR 0.35, 95% CI 0.21–0.58; *p* < 0.001). In contrast, high invasive-margin GLUT1 was independently associated with improved survival in the predefined solitary cohort (HR 0.379, 95% CI 0.18–0.81; *p* = 0.012). Double immunofluorescence demonstrated the qualitative co-localization of the GLUT1 signal with CD8⁺ cells at the infiltration margin. In exploratory assays (n = 5), flow cytometry suggested GLUT1 enrichment in CD8⁺ terminally differentiated effector memory T cells re-expressing CD45RA (TEMRA), and GLUT1⁺ TIL fractions showed higher in vitro tumor cell killing compared with GLUT1⁻ cells.

**Conclusion:**

GLUT1 shows compartment- and context-dependent associations in the CRLM. While tumor-core GLUT1 is linked to proliferative activity, invasive-margin GLUT1 is associated with favorable outcomes in solitary metastases. Immunofluorescence and functional data provide hypothesis-generating context and warrant validation with quantitative spatial immune profiling and independent cohorts.

**Supplementary Information:**

The online version contains supplementary material available at 10.1007/s00262-026-04378-z.

## Introduction

Colorectal cancer (CRC) is one of the most common malignancies worldwide and a leading cause of cancer-related deaths, with approximately 19 million new cases and over 900,000 deaths reported in 2020 alone [1]. The liver is the predominant site of distant metastasis, and colorectal liver metastases (CRLM) develop in up to 50% of patients during the course of the disease [2]. Among these, solitary CRLM constitutes a distinct clinical entity in which surgical resection offers a realistic chance of long-term survival and a potential cure.

Surgical resection of solitary CRLM provides the best chance for long-term survival, and patients with solitary lesions are often considered ideal surgical candidates [3, 4]. Nevertheless, recurrence rates after resection remain high, and long-term survival is far from guaranteed [5]. This discrepancy between technically successful surgery and heterogeneous oncologic outcomes highlights the need for biologically grounded prognostic markers and a deeper understanding of the mechanisms within the tumor microenvironment that influence patient outcomes.

The tumor microenvironment, particularly the immune landscape, plays a key role in CRC progression, including CRLM behavior. High densities of infiltrating CD8⁺ T cells are associated with improved survival [6–8], whereas the balance between effector and regulatory subsets can modulate tumor progression [9, 10]. Beyond cell density, the spatial organization of these lymphocytes, especially their accumulation at the invasive margin, has emerged as a key factor influencing patient outcomes and forms the basis of immune-based prognostic concepts, such as the Immunoscore [6–8]. However, the ability of these immune cells to mount an effective antitumor response critically depends on their metabolic fitness within the nutrient- and oxygen-deprived tumor microenvironment (TME). Similar to cancer cells, activated T lymphocytes rely on increased glucose uptake and glycolytic reprogramming to sustain their proliferation and effector functions.

Against this metabolic background, glucose transporter 1 (GLUT1) has been identified as a central mediator of cancer cell metabolism [11, 12]. GLUT1 is integral to the Warburg effect, a metabolic adaptation that enables cancer cells to sustain high rates of anaerobic glycolysis, even in the presence of oxygen. This metabolic shift is critical for supporting rapid proliferation and survival in a hostile tumor microenvironment, as highlighted by studies on its regulation via hypoxia-inducible factor-1 [13] and its overexpression in advanced cancers [12, 14]. It is crucial for meeting the energy demands of rapidly proliferating cancer cells and supporting their survival in the often harsh tumor microenvironment [15]. Elevated GLUT1 expression has been observed in various cancers, including CRC, and is associated with aggressive tumor behavior and poor prognosis [16, 17].

Beyond its role in tumor cells, GLUT1 is essential for activated T lymphocytes, enabling glucose uptake required for proliferation, differentiation, and effector functions [18, 19]. In this context, GLUT1 expression in T cells reflects metabolic engagement and functional activation rather than malignant transformation. Therefore, the presence of GLUT1-positive lymphocytes within the tumor microenvironment may indicate a metabolically competent antitumor response with potential prognostic relevance [6].

Despite growing interest in the metabolic and immune landscapes of colorectal cancer, the spatial metabolic interplay between tumor cells and infiltrating lymphocytes in colorectal liver metastases (CRLM) remains poorly defined. GLUT1 is a key mediator of glycolytic activity in both malignant cells and activated T cells; however, its compartment-specific relevance in CRLM remains unknown. Therefore, we hypothesized that spatially resolved GLUT1 expression may delineate distinct metabolic programs in tumor and immune compartments, and that these patterns could provide prognostic information beyond established clinicopathological markers.

## Material and methods

### Patient selection and tissue samples

This retrospective study included 192 consecutive patients who underwent curative-intent liver resection for CRLM at the University Hospital Regensburg (2004–2011). Clinical data and follow-up information were obtained from institutional databases. The CRLM diagnosis was confirmed pathologically after surgery. The study was approved by the Institutional Ethics Committee (no. 12–101-0009), and written informed consent was obtained from all patients.

The cohort comprised patients with solitary (n = 75) and multiple (n = 117) CRLM. Primary prognostic analyses were performed in a predefined subgroup of patients with solitary CRLM, while analyses in patients with multiple CRLM were considered exploratory and are labeled accordingly.

**Fig. 1:**
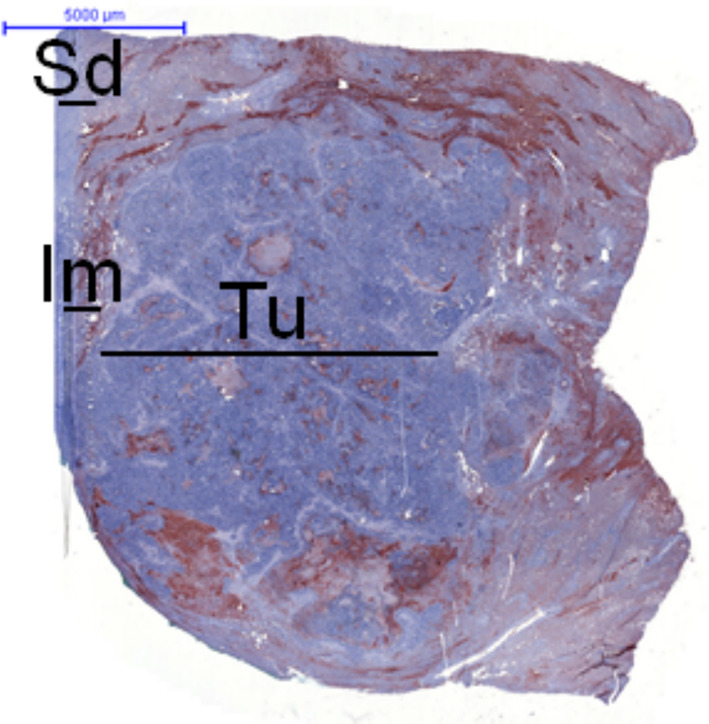
Orientation and compartment definitions for histologic assessment. A representative whole-section overview of a colorectal liver metastasis indicating the regions evaluated as tumor tissue (Tu), tumor–liver interface/infiltration margin (Im), and non-tumor tissue distant from the metastasis (Sd). This overview is provided to illustrate the compartmental approach; subsequent analyses were performed on representative high-power fields from the respective compartments, as described in the Methods section

FFPE tissue blocks from resected liver metastases were used for immunohistological analyses. For compartment-specific assessment, representative areas were evaluated in tumor tissue (Tu), at the tumor–liver interface/infiltration margin (Im), and in non-tumor tissue distant from the metastasis (Sd), as defined in the study. Sections were cut to a thickness of 5 µm for subsequent staining (Fig. 1).

### Immunohistochemistry

Immunohistochemical staining was performed on 5-µm FFPE sections. Deparaffinization was carried out by incubating the sections in Roti-Histol for 10 min twice, followed by rehydration through a graded ethanol series (99%, 96%, 90%, and 70%) and washing in fully deionized water. Heat-induced epitope retrieval was performed using citrate buffer. The sections were allowed to cool for 30 min and subsequently washed. Endogenous peroxidase activity was blocked using 0.3% hydrogen peroxide for 10 min. Blocking was performed using 5% goat serum for 1 h at room temperature.

The primary antibodies used for immunohistological analyses were GLUT1 (ab115730, Abcam), CD8 (ab17147, Abcam), and Ki67 (ab16667, Abcam). For Ki67 staining, the primary antibody was applied at a dilution of 1:100 and incubated overnight at 4 °C. Detection was performed using a biotinylated secondary antibody (biotinylated goat anti-rabbit IgG) followed by streptavidin–peroxidase, and visualization was achieved with a DAB chromogen (Zytomed Systems, Berlin, Germany). Slides were counterstained with hematoxylin and subsequently mounted.

### Assessment of GLUT1 and Ki67

GLUT1 expression intensity was semi-quantitatively assessed and categorized as high or low within each compartment (Tu, Im, Sd). Each slide was evaluated twice, and discrepant ratings were adjudicated by a third reviewer. Ki67 was quantified by manual counting of Ki67-positive nuclei at 20 × magnification, assessed separately for tumor and stromal compartments. For dichotomized analyses, samples were classified as Ki67-high or Ki67-low using the median number of Ki67-positive cells per 20 × field of view as the cut-off.

### Double immunofluorescence staining for CD8 and GLUT1

Double immunofluorescence staining was performed on a representative FFPE section. After deparaffinization and rehydration, heat-induced antigen retrieval was carried out in TRIS–EDTA buffer using a steamer (pre-heated retrieval solution), followed by cooling to room temperature. Sections were washed in T-TBS, delineated with a hydrophobic barrier pen, and blocked with 5% goat serum for 1 h at room temperature. Slides were incubated overnight at 4 °C with anti-GLUT1 (1:750; Abcam, Cambridge, UK; ab115730), washed three times in T-TBS (5 min each), and incubated with goat anti-rabbit Cy3 (1:200; Dianova, Hamburg, Germany; 111-165-045) for 1 h at room temperature. After three additional washes in T-TBS, slides were incubated overnight at 4 °C with anti-CD8 (1:50; Abcam, Cambridge, UK; ab17147), washed three times in T-TBS (5 min each), and incubated with goat anti-mouse Cy2 (1:200; Dianova, Hamburg, Germany; 115-225-146) for 1 h at room temperature. Nuclei were counterstained with DAPI and sections were mounted using fluorescent mounting medium (Dako). Images were acquired on an Axio Observer Z1 fluorescence microscope (Zeiss) at 40 × magnification using appropriate fluorescence filters. Co-localization was assessed qualitatively; no quantitative image analysis was performed.

### Flow cytometry

Flow cytometry was performed on two sets of samples: peripheral blood mononuclear cells (PBMCs) obtained preoperatively from 19 patients and tumor-derived leukocytes isolated from fresh resection specimens (tumor–liver interface) of five patients. PBMCs were used to characterize the systemic immune phenotype, whereas tumor-derived leukocytes were analyzed to explore the local immune infiltrate at the tumor–liver interface. Mononuclear cells were isolated by density-gradient centrifugation using Ficoll-Paque PLUS (GE Healthcare, Marlborough, MA, USA). Viability was assessed using Fixable Viability Dye eFluor 506 (eBioscience, San Diego, CA, USA), and non-viable cells were excluded. Surface staining was performed using fluorochrome-conjugated antibodies against CD3, CD4, and CD8 (BD Biosciences, Franklin Lakes, NJ, USA) and GLUT1 (R&D Systems, Minneapolis, MN, USA). Data were acquired using a FACSCanto II flow cytometer (BD Biosciences) and analyzed using FlowJo v10.8 (BD, Ashland, OR, USA). The gating strategy comprised the selection of CD45-positive leukocytes, lymphocytes, CD3-positive T cells, and subsequent subdivision into CD4-positive and CD8-positive subsets. GLUT1 expression was quantified within each subset. Results are presented as the mean ± standard deviation.

### Killing assay

The cytotoxic activity of tumor-infiltrating lymphocytes (TILs) was assessed in co-culture assays using the SW480 colorectal cancer cell line as the target. TILs were separated into GLUT1-positive and GLUT1-negative fractions by magnetic-activated cell sorting (MACS; Miltenyi Biotec, Bergisch Gladbach, Germany). Effector cells were co-cultured with target cells at an effector-to-target ratio of 20:1. Cytotoxicity was quantified by lactate dehydrogenase (LDH) release into culture supernatants using a Cytotoxicity Detection Kit (Roche, Basel, Switzerland) and by fluorescence-based microscopic visualization of cell death using YoYo-Green staining (Thermo Fisher Scientific, Waltham, MA, USA). For LDH measurements, spontaneous release (target cells alone) and maximum release (lysis with Triton X-100) were included as controls.

Specific lysis percentages were calculated as follows:

$$Specific\;Lysis \left(\%\right)= \frac{Experimental\;LDH\;relase-Spontaneus\;LDH\;release}{Maximum\;LDH\;release-Spontaneus\;LDH\;release} x 100$$

All experiments were performed in triplicate using TILs isolated from five patients. Owing to the small sample size, the results are reported as exploratory. Statistical analysis was conducted to compare cytotoxic capacities across groups, with significance set at *p* < 0.05. Prior to each experimental series, cell cultures were tested for mycoplasma contamination using a PCR-based assay, and only mycoplasma-negative cultures were used. STR-based cell line authentication certificates were not available for the historic workflow.

### Statistical analysis

Statistical analyses were performed using IBM SPSS Statistics for Windows, version 29.0 (IBM Corp., Armonk, NY, USA). Continuous variables are reported as mean ± SD or median with interquartile range (IQR), as appropriate. For dichotomized analyses, GLUT1 and Ki67 were classified into high-risk and low-risk groups using cohort median cut-offs based on the respective assessment method (semi-quantitative GLUT1 score; Ki67 quantified by manual counting/labeling index). Overall survival (OS) was defined as the interval between liver resection and death from any cause. Survival distributions were estimated using the Kaplan–Meier method and compared using the log-rank test. Cox proportional hazards regression was used for univariable and multivariable analyses, reporting hazard ratios (HR) with 95% confidence intervals (CI). Multivariable models were adjusted for age, sex, primary tumor location, and perioperative chemotherapy. All tests were two-sided, and statistical significance was set at *P* < 0.05.

## Results

### Spatial assessment of GLUT1 across tumor and peritumoral compartments

A total of 192 patients who underwent curative-intent liver resection for colorectal liver metastases (CRLM) were included in this study. The baseline clinicopathological characteristics are summarized in Table 1. The median follow-up duration was 39 months (interquartile range, 15–72).

**Table 1:** Baseline characteristics of patients who underwent curative-intent resection for colorectal liver metastases (N = 192)

Characteristic	n (%)
Age, median (IQR)	61 (53–68)
*Sex*	
Male	112 (58.3)
Female	80 (41.7)
*Primary tumor location*	
Colon	116 (60.4)
Rectum	76 (39.6)
*Metastatic burden*	
Solitary	75 (39.1)
Multiple	117 (60.9)
*Timing of metastasis*	
Synchronous	84 (43.8)
Metachronous	108 (56.2)
*Adjuvant chemotherapy*	
Yes	78 (40.6)
No	114 (59.4)
*Pathologic T stage*	
T1	6 (3.1)
T2	23 (12.0)
T3	122 (63.5)
T4	38 (19.8)
Tx / unknown	3 (1.6)
*Pathologic N stage*	
N0	67 (34.9)
N1	63 (32.8)
N2	59 (30.7)
Nx / unknown	3 (1.6)
*Tumor grade* (*G*)	
G1	2 (1.0)
G2	147 (76.6)
G3	41 (21.4)
Gx/unknown	2 (1.0)
*Resection margin status*	
R0	161 (83.9)
R1	31 (16.1)
*Capsule status*	
Present	75 (39.1)
Absent	117 (60.9)

Continuous variables are reported as median (interquartile range [IQR]) and range, and categorical variables as n (%). Percentages are calculated using the total cohort size

The cohort comprised patients with solitary (n = 75) and multiple (n = 117) CRLM. Primary prognostic analyses were performed in a predefined subgroup of patients with solitary CRLM, whereas analyses including patients with multiple CRLM were considered exploratory and are labeled accordingly in the respective tables and figure legends.

A schematic overview of the compartmental approach (tumor tissue, tumor–liver interface/infiltration margin, and distant non-tumor tissue; Tu/Im/Sd) is provided in Fig. 1. Representative CD8/GLUT1 double immunofluorescence images illustrating qualitative colocalization at the infiltration margin are shown in Supplementary Fig. S4.

### Influence of GLUT1 expression on patient survival

**Fig. 2:**
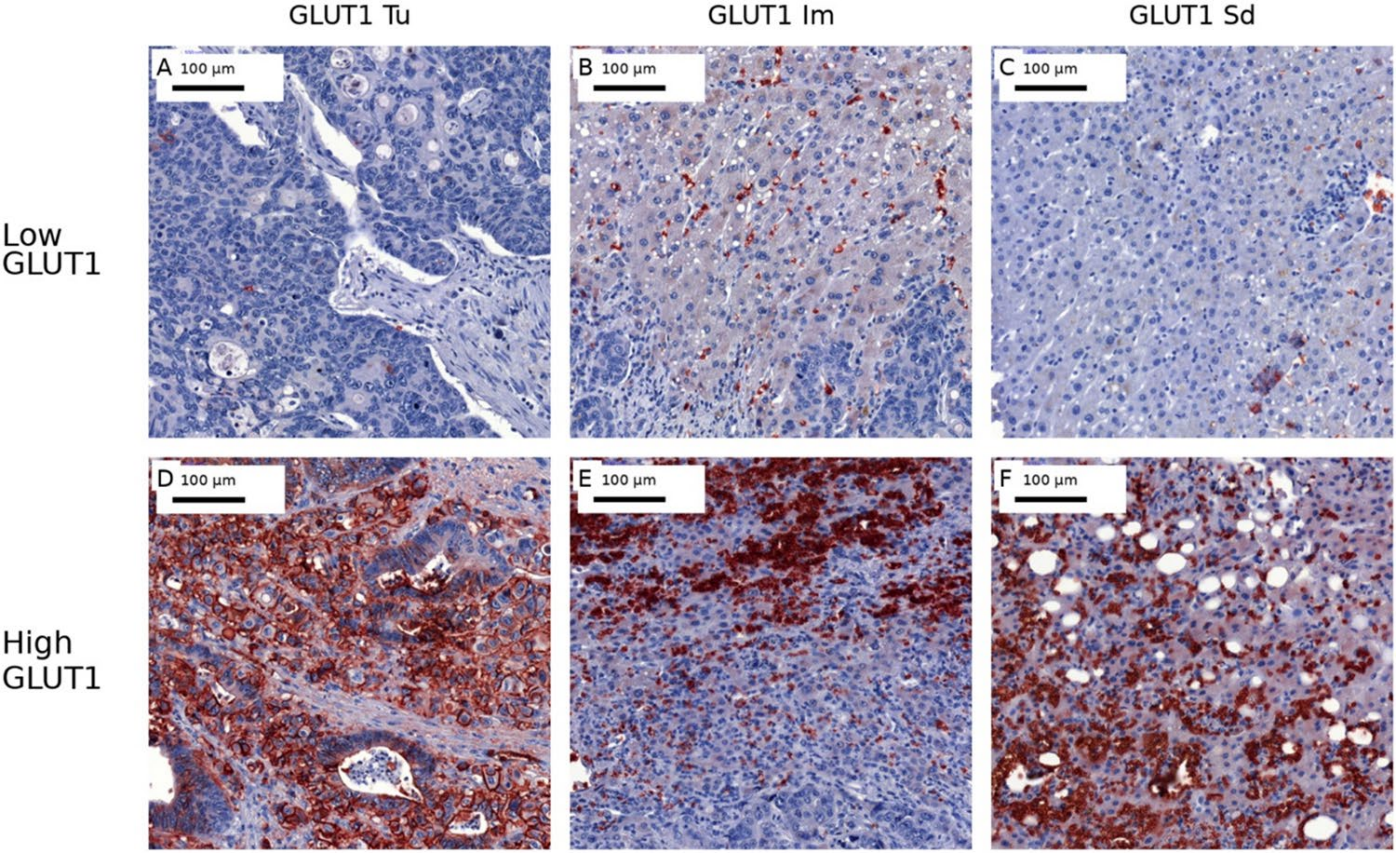
Immunohistochemical staining of GLUT1-positive cells in the CRLM across spatial compartments. Representative 20 × images show GLUT1 staining in the tumor tissue (Tu), infiltration margin defined as the liver tissue directly adjacent to the tumor (Im), and healthy liver tissue (Sd). Cases were semi-quantitatively categorized as GLUT1 low (A–C) or GLUT1 high (D–F) within each compartment (Tu: A/D; Im: B/E; Sd: C/F). Scale bar, 100 µm

High GLUT1 expression was observed in 49.5% of tumor cores (Tu), 60.8% of invasive margins (Im), and 51.1% of surrounding liver tissue (Sd; Fig. 2). 2).

**Fig. 3:**
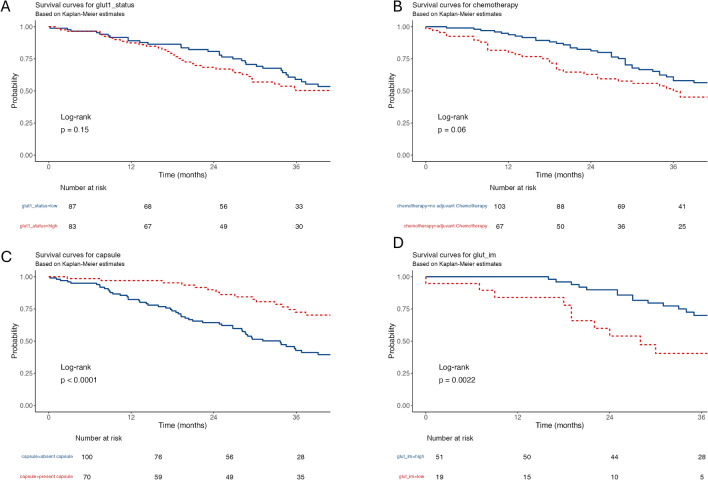
Kaplan–Meier survival analysis based on GLUT1 expression in CRLM. **A** Overall survival according to tumor GLUT1 expression levels. **B** Overall survival stratified by adjuvant chemotherapy (yes vs. no). **C** Overall survival in patients with and without a fibrous capsule. **D** Subgroup analysis of patients with solitary metastases showing overall survival according to invasive-margin GLUT1 expression (high vs. low). Log-rank p-values are shown for each comparison. The number-at-risk tables are presented beneath the corresponding curves

Kaplan–Meier analyses were restricted to patients with complete overall survival (OS) data (n = 170). In this survival-complete cohort, tumoral GLUT1 did not discriminate OS (Fig. 3A; log-rank *p* = 0.151). By contrast, capsule status clearly separated the curves, with markedly longer survival in patients with a fibrous capsule (Fig. 3C; log-rank *p* < 0.001). IM-GLUT1 showed only a borderline association in the overall cohort (log-rank *p* = 0.057), and adjuvant chemotherapy was similarly associated with a borderline shift towards poorer OS (Fig. 2B; log-rank *p* = 0.060).

Capsule-stratified analyses were performed in a predefined solitary cohort (n = 70) to assess whether IM-GLUT1 merely reflected capsule status. Within capsule-absent cases (n = 40), IM-GLUT1 high was associated with longer OS (median 49 vs. 22 months; log-rank *p* = 0.037) and a reduced hazard in univariable Cox regression (HR 0.40, 95% CI 0.17–0.98; *p* = 0.045). A similar separation was observed among capsule-present cases (n = 30), where the median OS was not reached in the IM-GLUT1-high group (log-rank *p* = 0.037); Cox estimates suggested a protective association, although precision was limited by small numbers in the IM-GLUT1-low group (n = 6; HR 0.28, 95% CI 0.08–1.00; *p* = 0.050). In contrast, capsule-stratified analyses in the overall OS-complete cohort (n = 170) did not show statistically significant associations between IM-GLUT1 and OS within either capsule stratum and were considered exploratory.

Primary prognostic analyses were predefined for solitary CRLM. Among the 70 solitary cases with complete OS data, IM-GLUT1 was strongly associated with outcome (Fig. 3D; log-rank *p* = 0.002). In the univariable Cox regression, IM-GLUT1 high versus low was linked to a substantially reduced hazard of death (HR 0.338, 95% CI 0.16–0.70; *p* = 0.003) (Table 2). The association persisted after adjustment (adjusted HR 0.379, 95% CI 0.18–0.81; *p* = 0.012).

**Table 2:** Univariable and multivariate Cox proportional hazards regression analyses for overall survival

Variable	Level (comparison)	HR (univariable)	*p* (univariable)	HR (multivariable)	*p* (multivariable)
*Main cohort*					
Tumoral GLUT1	High versus low	1.38 (0.89–2.15)	0.152	1.183 (0.74–1.90)	0.485
Adjuvant chemotherapy	Yes versus no	1.52 (0.98–2.36)	0.062	1.494 (0.92–2.41)	0.101
Capsule	Present versus absent	0.34 (0.20–0.57)	< 0.001	0.35 (0.21–0.58)	< 0.001
Invasive-margin GLUT1	High versus low	0.65 (0.41–1.02)	0.060	0.693 (0.43–1.11)	0.127
*Subgroup: solitary metastases*					
Invasive-margin GLUT1	High versus low	0.34 (0.16–0.70)	0.003	0.379 (0.18–0.81)	0.012

Cox models were fitted as complete-case analyses. The overall-cohort multivariate model includes patients with available OS time/event and covariates (n = 170). The predefined solitary cohort analysis includes solitary CRLM with complete OS data (n = 70). Hazard ratios (HR) are shown with 95% confidence intervals

Multivariable Cox models in the overall cohort were fitted as complete case analyses (n = 170). Capsule presence remained independently prognostic (adjusted HR 0.355, 95% CI 0.21–0.60; *p* < 0.001). Neither tumoral GLUT1 (adjusted HR 1.183, 95% CI 0.74–1.90; *p* = 0.485) nor IM-GLUT1 (adjusted HR 0.693, 95% CI 0.43–1.11; *p* = 0.127) retained statistical significance after adjustment. The same was true for adjuvant chemotherapy (adjusted HR 1.494, 95% CI 0.92–2.41; *p* = 0.101) (Table 2).

### Association with proliferation marker Ki67

**Fig. 4:**
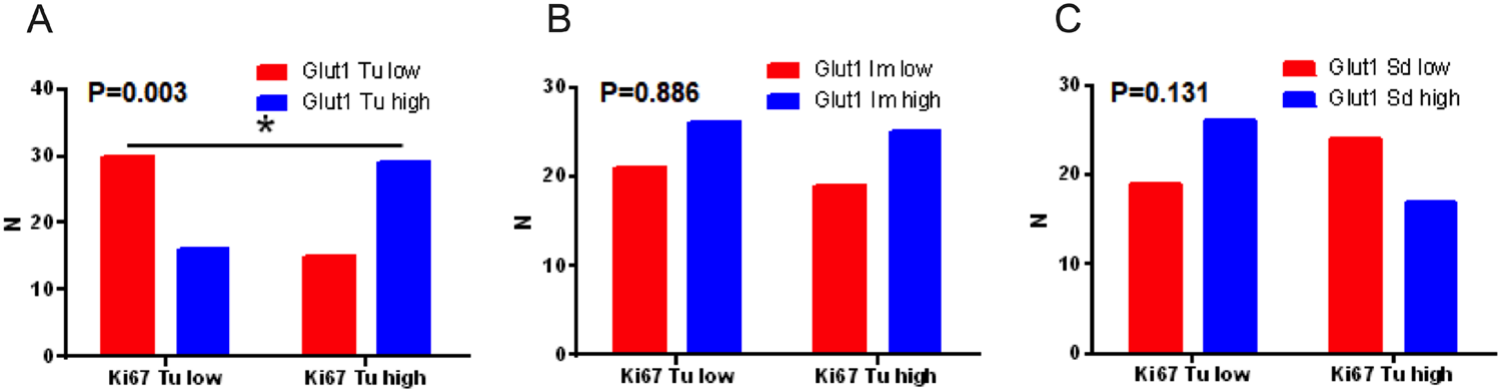
Association between the Ki67 proliferation index and GLUT1 expression in distinct CLM regions. **A** Immunohistochemical analysis of 93 patient samples revealed a significant correlation between high Ki67 expression and GLUT1-positivity in the tumor core (*p* = 0.003), **B** but not at the invasive margin (*p* = 0.886) or **C** in adjacent liver tissue (*p* = 0.131). These findings suggest that GLUT1 expression in the tumor core is associated with increased cellular proliferation

In a subset of 93 patients, Ki67 expression was assessed in the tumor, invasive margin, and surrounding liver tissue. Ki67 expression positively correlated with GLUT1 expression in the tumor core (Spearman’s ρ = 0.31, *p* = 0.003). No significant associations were detected at the invasive margin (*p* = 0.886) or in the surrounding liver tissue (*p* = 0.131) (Fig. 4).

### Flow cytometric analysis of GLUT1-positive immune cells in CRLM

Flow cytometric analysis of leukocytes isolated from the tumor–liver transition zone was performed in five CRLM specimens. Among the CD45⁺ cells, 37.4 ± 9.2% expressed GLUT1. The highest proportion of GLUT1⁺ cells was observed in monocytes (81.5 ± 14.8%) and granulocytes (68.0 ± 17.0%). Within the lymphocyte population, CD8⁺ T cells were more frequent than CD4⁺ T cells (59.9 ± 9.7% vs. 32.4 ± 12.9%), and GLUT1 expression was enriched within the CD8⁺ subset. Subdivision of CD8⁺ T cells revealed that terminally differentiated effector memory T cells re-expressing CD45RA (TEMRA) cells exhibited the highest levels of GLUT1 (40.4 ± 5.0%). Given the limited sample size (n = 5), these findings should be considered exploratory.

### Cytotoxic activity of GLUT1-positive lymphocytes

The cytotoxic activity of GLUT1⁺ versus GLUT1⁻ lymphocytes was evaluated in co-culture assays using SW480 colorectal cancer cells. GLUT1⁺ TILs exhibited enhanced cytotoxicity compared to their GLUT1⁻ counterparts, as measured by LDH release and confirmed by YoYo-Green microscopy (n = 5). Appropriate controls for spontaneous and maximum lysis were also included. These findings indicate that GLUT1⁺ lymphocytes display increased tumor cell-killing capacity; however, the results should be interpreted as exploratory due to the limited number of samples (Figure S1).

### Cytotoxicity of TEMRA cells

Within the CD8⁺ compartment, GLUT1⁺ TEMRA cells were further characterized using functional flow cytometry. GLUT1⁺ TEMRA cells showed higher expression of Granzyme B than GLUT1⁻ TEMRA cells (*p* = 0.062, trend), while no significant differences were observed for CD107a, IFNγ, or Perforin (all n.s.). Additional markers, including IL-33 and FasL, could not be reliably evaluated due to insufficient separation. Taken together, these results suggest a trend toward enhanced effector function in GLUT1⁺ CD8⁺ TEMRA cells; however, the small sample size (n = 5) limits definitive conclusions (Figure S2).

## Discussion

In this translational study, we investigated the prognostic impact of spatially resolved GLUT1 expression in CRLM and explored its relationship to immune–tumor interface biology in CRC [1, 9, 17]. The immune system plays a critical role in the recognition and elimination of malignant cells, which has driven interest in understanding immune–tumor interactions in CRC and its metastases [20]. Against this background, we investigated the prognostic impact of GLUT1 expression in CRLM, with a particular focus on its spatial localization within the tumor microenvironment.

In our cohort, tumor GLUT1 did not emerge as a statistically significant prognostic marker, despite a directionally adverse association with overall survival. This finding contrasts with earlier reports linking high GLUT1 expression to metabolically aggressive colorectal cancer biology [11, 12, 16, 17] and suggests that within established liver metastases, GLUT1 expression may reflect proliferative activity without providing independent prognostic information. Consistent with this interpretation, tumoral GLUT1 did not retain statistical significance after multivariate adjustment, suggesting a limited incremental prognostic value of tumor-core GLUT1 in this setting.

In contrast, high GLUT1 expression at the invasive margin was associated with improved survival in patients with solitary metastases. This favorable association is compatible with prior evidence that activated immune effector cells can upregulate GLUT1 and may display enhanced cytotoxic programs [18–22] and with previous studies underscoring the prognostic importance of spatially organized CD8+ lymphocytes at metastatic interfaces [6–10]. Together, these observations are consistent with a spatially context-dependent prognostic signal at the tumor–liver interface, but do not establish a causal mechanism [23, 24].

In addition to GLUT1-related parameters, the capsular growth pattern remained a robust prognostic marker, potentially reflecting a coordinated stromal and immune response rather than a purely anatomical feature. This interpretation is consistent with recent literature indicating that fibrotic or capsule-like interfaces reflect host-mediated containment of metastatic [25, 26]. Evidence from systematic analyses further supports the view that capsule-like or fibrotic growth patterns reflect a more contained mode of metastatic expansion, consistent with host-mediated stromal and immune responses26. In this context, capsular structures may define a microenvironment that restricts tumor expansion and shapes the metabolic demands and spatial organization of infiltrating immune cells, potentially including GLUT1-expressing CD8⁺ T cells. To address whether IM-GLUT1 merely reflects capsule status, we performed capsule-stratified analyses in the solitary OS-complete cohort. IM-GLUT1 remained associated with improved survival within both capsule-absent and capsule-present strata, although estimates in the capsule-present/IM-GLUT1-low subgroup were imprecise owing to small sample sizes. In the overall OS-complete cohort, capsule-stratified analyses did not show statistically significant associations. Exploratory interaction analyses in the solitary cohort did not provide definitive evidence that the IM-GLUT1 association is modified by IL-17/IFN-γ interface measures or by CD4Sn, while a borderline trend for interaction with CD8Sn was observed.

The positive correlation between tumoral GLUT1 and the proliferation marker Ki67 (Spearman ρ = 0.31, *p* = 0.003) further supports the interpretation that tumoral GLUT1 reflects aggressive and proliferative tumor biology. No such association was observed at the invasive margin. This pattern is compatible with a non–tumor-cell contribution to the IM-GLUT1 signal, but cell-type attribution at the interface remains limited by the semi-quantitative scoring approach and qualitative immunofluorescence [27]. This distinction between tumor-core and interface-associated GLUT1 signals is important for interpreting spatially resolved prognostic associations.

Our exploratory functional analyses provide a biologically plausible context but do not establish a mechanism. Flow cytometry suggested enrichment of GLUT1 expression in CD8⁺ T cells, including the TEMRA subset, and GLUT1⁺ lymphocytes showed higher cytotoxicity in vitro [28, 29]. GLUT1⁺ TEMRA cells displayed higher levels of Granzyme B than GLUT1⁻ TEMRA cells (*p* = 0.062, trend), whereas no significant differences were observed for CD107a, IFNγ, or Perforin [30]. Although limited by sample size (n = 5) and therefore exploratory in nature, these findings are compatible with the hypothesis that GLUT1 may mark metabolically active effector T cells with enhanced antitumor capacity at the invasive margin [18, 19]. Therefore, these functional and flow cytometric data should be viewed as generating a mechanistic hypothesis that may explain the clinical associations observed in a larger cohort, rather than providing definitive proof of a causal link between GLUT1 + CD8+ T cells and improved survival.

In summary, these results suggest that the prognostic relevance of GLUT1 in CRLM is strongly dependent on the spatial and clinical context. Tumor-associated GLUT1 expression did not demonstrate independent prognostic value in this cohort, whereas invasive-margin GLUT1 was associated with improved survival, specifically in the predefined solitary cohort. This pattern suggests a context-dependent biological role rather than a uniform adverse or favorable effect [23, 24]. These observations underscore the importance of spatial context in biomarker evaluation and may explain discrepancies in the literature regarding the prognostic significance of GLUT1 in CRC [11, 12, 16].

From a clinical perspective, spatial GLUT1 assessment could provide complementary information to established prognostic markers. In particular, the association of margin-associated GLUT1 with favorable outcomes resembles the concept of the immunoscore, which highlights the prognostic value of immune infiltration at the invasive margin. However, independent validation and quantitative spatial immune profiling are required before clinical implementation.

Systemic chemotherapy is an important potential confounder in our analysis. In our verified multivariate models, adjuvant chemotherapy did not retain independent statistical significance, despite borderline associations in univariable and Kaplan–Meier analyses. This likely reflects the selection bias inherent to retrospective studies, as patients with more aggressive tumor biology, higher disease burden, or adverse clinical features are more likely to receive adjuvant treatment. Moreover, detailed information on chemotherapy regimens, dose intensity, treatment response, and toxicity was not available in a standardized manner. Chemotherapeutic agents can modulate both tumor and immune cell metabolism, including glycolytic pathways and GLUT1 expression. Consequently, the observed associations between spatial GLUT1 patterns and outcomes must be interpreted with caution in the context of treatment heterogeneity. Prospective studies with defined sampling time points and comprehensive therapy annotation will be necessary to disentangle treatment-related from intrinsic immunometabolic effects.

This study has several limitations that should be acknowledged. First, this was a retrospective single-center study, which may limit generalizability and introduces the risk of residual confounding. Second, the compartment-based assessment at the infiltration margin was field-based and not defined by a fixed millimeter distance from the tumor border; intralesional heterogeneity may therefore introduce sampling bias. Third, GLUT1 was assessed semi-quantitatively as high versus low; despite repeated reads, alternative thresholds or quantitative scoring approaches might yield different results. Fourth, mechanistic inference is limited: double immunofluorescence was evaluated qualitatively, and we did not perform quantitative spatial image analysis of CD8⁺GLUT1⁺ cell density across the cohort; consequently, we cannot formally demonstrate incremental prognostic value of IM-GLUT1 beyond immune cell density or other spatial immune metrics. Fifth, the functional analyses (flow cytometry and killing assays) were exploratory and based on a very small sample size (n = 5). The killing assay is further limited by the use of a single, non–patient-matched CRC cell line and a high effector-to-target ratio (20:1), and potential non-specific/alloreactive killing cannot be excluded. Finally, systemic therapy effects on GLUT1 and immune phenotypes could not be addressed in a regimen-resolved manner; we did not perform external validation, systematic histopathological growth pattern classification beyond capsule status, or extended metabolic IHC panels (e.g., LDHA/MCT4). These aspects should be addressed in future multi-center studies with quantitative spatial immune profiling and independent validation cohorts.

In summary, our data support a context-dependent association between spatial GLUT1 patterns and outcome in CRLM. Tumor-core GLUT1 did not provide independent prognostic information, whereas high invasive-margin GLUT1 was associated with improved survival in the predefined solitary cohort; the cellular contributors to the interface signal remain to be quantified at the cohort level. Capsule status emerged as the strongest prognostic factor across all models, reflecting a host-mediated containment pattern that integrates stromal and immune responses. As an external context, we queried a public CRLM single-cell atlas and observed SLC2A1 signals across multiple author-defined lineages, including CRC cells, CD8+ T cells, macrophages, and endothelial cells (Supplementary Fig. S3). Beyond surgical prognostication, these findings motivate independent validation and quantitative spatial immune profiling to clarify whether IM-GLUT1 adds prognostic information beyond established clinicopathological predictors and immune density.

## Supplementary Information

Below is the link to the electronic supplementary material.

Supplementary file1 (DOCX 2840 KB)

## Data Availability

The data underlying this study cannot be shared publicly because of privacy and ethical restrictions. De-identified data and analytical methods will be made available upon reasonable request to the corresponding author to reproduce the results or replicate the procedures.
