# Supplementary material for: Compartment-specific GLUT1 patterns in colorectal liver metastases: invasive-margin GLUT1 associates with outcome in solitary disease
Source: Cancer Immunol Immunother. 2026 Apr 6;75(5):135. doi: 10.1007/s00262-026-04378-z (PMC13053725; doi:10.1007/s00262-026-04378-z)
Supplement: Supplementary file 1 — Supplementary file1 (DOCX 2840 KB) [file 262_2026_4378_MOESM1_ESM.docx]

**Supplementary Material**


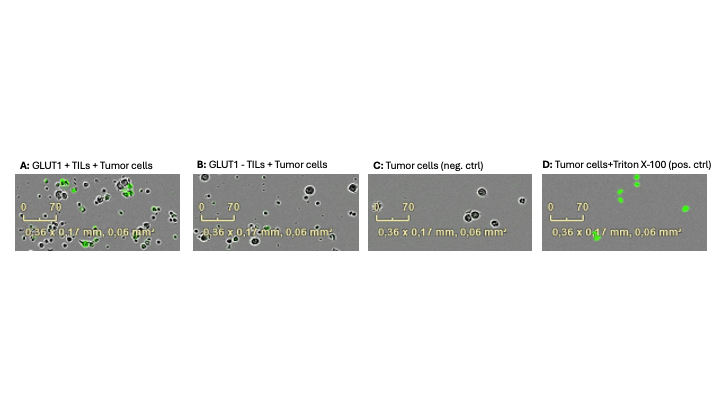


**Figure S1: In vitro cytotoxicity assay demonstrating enhanced tumor cell killing by GLUT1-positive tumor-infiltrating lymphocytes (TILs).** YoYo-green staining indicates apoptotic tumor cells after 3-hour co-culture.
(A)GLUT1^+^ TILs show marked cytotoxic activity against colorectal cancer cells.
(B)GLUT1 ^–^ TILs exhibit minimal cytotoxicity under identical conditions.
(C)Negative control: tumor cells cultured alone show negligible cell death.
(D)Positive control: tumor cells lysed with Triton X-100 show extensive cell death.
Cell density and lysis are quantified in each panel (cells/mm^2^).


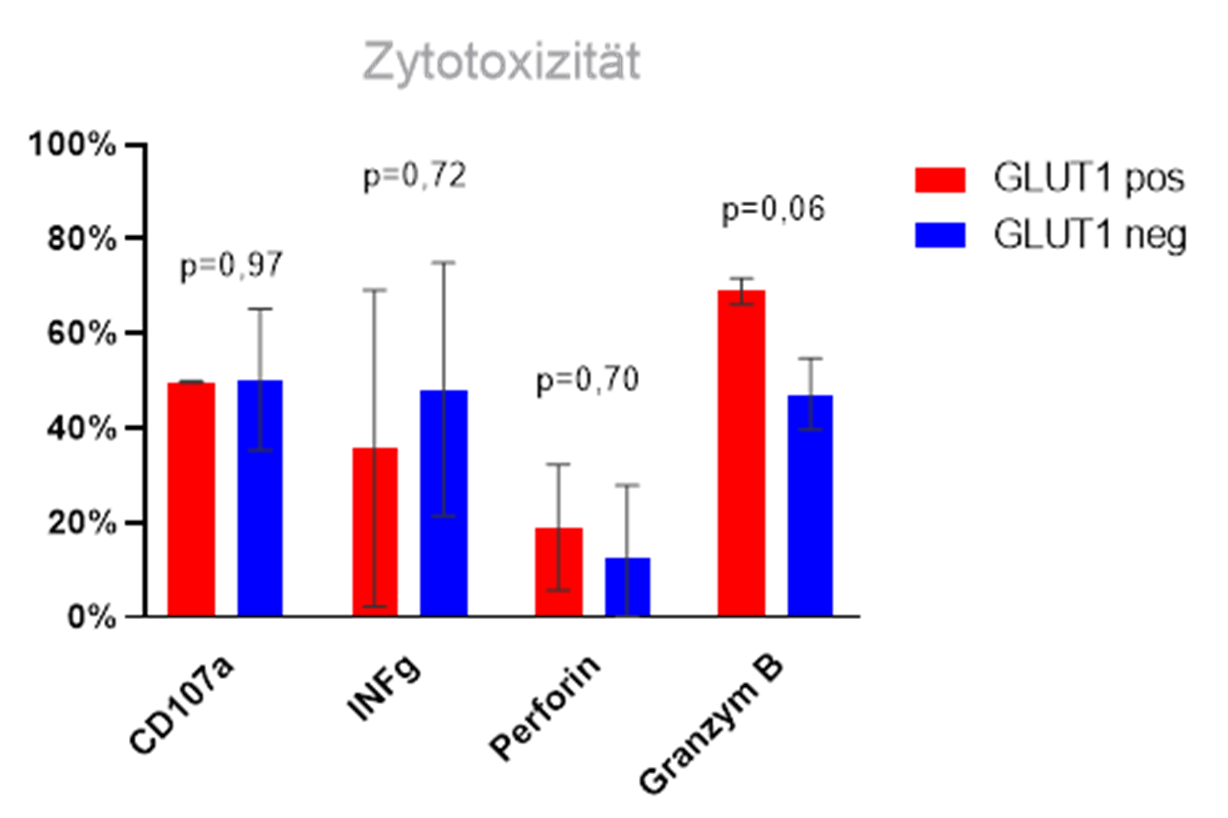


**Figure S2: Functional marker expression in CD8^+^ TEMRA cells stratified by GLUT1 expression.**
Flow cytometric analysis of CD8^+^ TEMRA cells from CRLM revealed increased Granzyme B expression in GLUT1^+^ compared to GLUT1^-^ cells. No significant differences were observed in IFN-y or perforin expression between the two subpopulations.


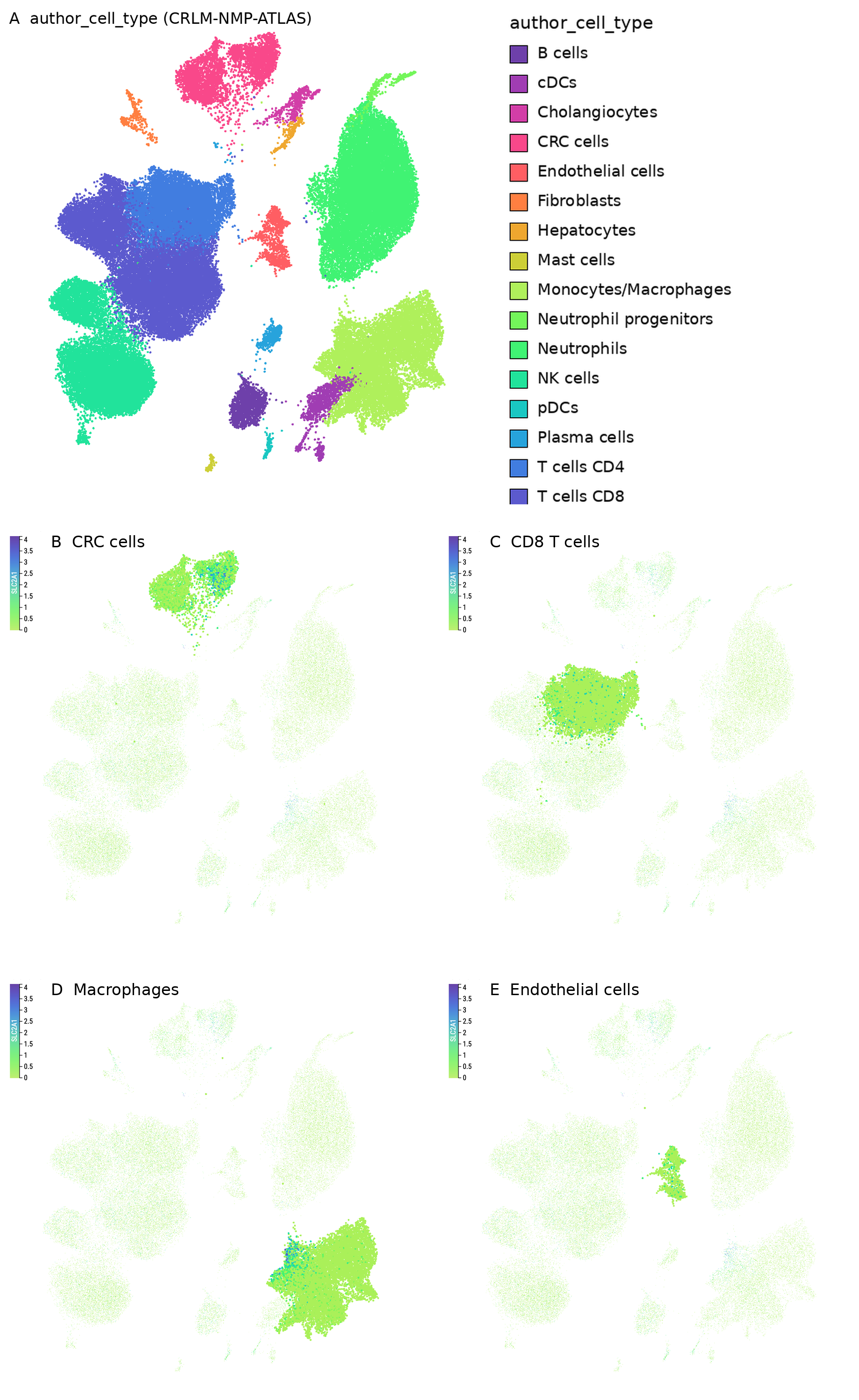


**Figure S3: Public single-cell atlas query of SLC2A1 (GLUT1) in colorectal liver metastases.**

UMAP embedding from the CRLM-NMP-ATLAS dataset visualized in the CELLxGENE Explorer. (A) UMAP colored by author-defined cell types. Cell type–restricted views are shown for qualitative context, highlighting SLC2A1 expression patterns within selected lineages: (B) CRC cells, (C) CD8 T cells, (D) macrophages, and (E) endothelial cells. Panels are colored by normalized SLC2A1 expression using the CELLxGENE scale (0–4). No quantitative downstream single-cell reanalysis was performed.


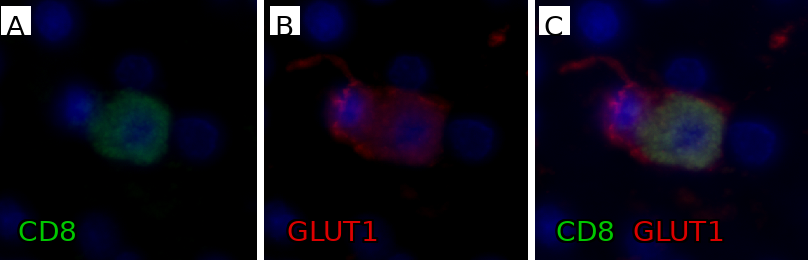


**Supplementary Figure S4. Representative CD8/GLUT1 double immunofluorescence at the tumor–liver interface.** Representative immunofluorescence images showing CD8 (green) and GLUT1 (red) signals in tumor-infiltrating lymphocytes at the infiltration margin. (A) CD8 channel, (B) GLUT1 channel, and (C) merged image demonstrating spatial co-localization. Nuclei are counterstained with DAPI (blue). Images are provided for qualitative visualization; no quantitative image analysis was performed.
